# Differences Between High vs. Low Performance Chess Players in Heart Rate Variability During Chess Problems

**DOI:** 10.3389/fpsyg.2019.00409

**Published:** 2019-02-26

**Authors:** Juan P. Fuentes-García, Santos Villafaina, Daniel Collado-Mateo, Ricardo de la Vega, Pedro R. Olivares, Vicente Javier Clemente-Suárez

**Affiliations:** ^1^Faculty of Sport Science, University of Extremadura, Cáceres, Spain; ^2^Facultad de Educación, Universidad Autónoma de Chile, Talca, Chile; ^3^Department of Physical Education, Sport and Human Movement, Autonomous University of Madrid, Madrid, Spain; ^4^Faculty of Education, Psychology and Sport Sciences, University of Huelva, Huelva, Spain; ^5^Faculty of Sports Sciences, Universidad Europea de Madrid, Madrid, Spain; ^6^Grupo de Investigación Cultura, Educación y Sociedad, Universidad de la Costa, Barranquilla, Colombia

**Keywords:** autonomic modulation, chess, cognition, heart rate variability, cognitive load

## Abstract

**Background:** Heart rate variability (HRV) has been considered as a measure of heart-brain interaction and autonomic modulation, and it is modified by cognitive and attentional tasks. In cognitive tasks, HRV was reduced in participants who achieved worse results. This could indicate the possibility of HRV predicting cognitive performance, but this association is still unclear in a high cognitive load sport such as chess.

**Objective:** To analyze modifications on HRV and subjective perception of stress, difficulty and complexity in different chess problem tasks.

**Design:** HRV was assessed at baseline. During the chess problems, HRV was also monitored, and immediately after chess problems the subjective stress, difficulty and complexity were also registered.

**Methods:** A total of 16 male chess players, age: 35.19 (13.44) and ELO: 1927.69 (167.78) were analyzed while six chess problem solving tasks with different level of difficulty were conducted (two low level, two medium level and two high level chess problems). Participants were classified according to their results into two groups: high performance or low performance.

**Results:** Friedman test showed a significant effect of tasks in HRV indexes and perceived difficulty, stress and complexity in both high and low performance groups. A decrease in HRV was observed in both groups when chess problems difficulty increased. In addition, HRV was significantly higher in the high performance group than in the low performance group during chess problems.

**Conclusion:** An increase in autonomic modulation was observed to meet the cognitive demands of the problems, being higher while the difficulty of the tasks increased. Non-linear HRV indexes seem to be more reactive to tasks difficulty, being an interesting and useful tool in chess training.

## Introduction

The game of chess has been traditionally used for the study of basic cognitive processes (memory or problem solving) (Amidzic et al., 2006; Troubat et al., 2009) where the executive function plays an important role (Elkies and Stanley, 2003). Gobet and Simon (1998) suggested that expert chess players have a large database of chunks (Chase and Simon, 1973) stored in their long-term memory. This database can be used as working memory, increasing the processing capacities of chess players (Guida et al., 2012). This is supported by complex visual processing outside of conscious awareness (Kiesel et al., 2009). Moreover, skills as logic, intellectual capacity and mathematical problem-solving are required in chess players (Aciego et al., 2012; Kazemi et al., 2012; Bart, 2014; Lin et al., 2015; Mathy et al., 2016; Sala et al., 2017). Chess has been proposed as a useful tool to improve mathematical problem-solving abilities due to the cognitive processes involved in the game (Kazemi et al., 2012; Sala et al., 2015).

One of the most relevant functions of the prefrontal cortex is the decision-making. This process is extremely relevant in chess because players always have to plan and decide their next move (Koechlin and Hyafil, 2007). The prefrontal cortex is associated with the vagal function, which is easily measured by heart rate variability (HRV) (variation in the beat-to-beat interval) (Thayer et al., 2012). There is a dynamic balance between sympathetic and the parasympathetic nervous systems (autonomic modulation). Parasympathetic activity, which leads to an increment in the HRV, is frequent at rest and in relaxing situation. Sympathetic activity is related with stressful situations and leads to a reduction in the HRV (Shaffer et al., 2014).

HRV has been considered as a measure of heart-brain interaction (Shaffer et al., 2014), and it is also modified by cognitive, attentional tasks or anxiogenic response (Porges and Raskin, 1969; Goldman-Rakic, 1996; Shinba et al., 2008; Mukherjee et al., 2011). An increase in sympathetic modulation analyzed in time, frequency and nonlinear domains was observed when cognitive demand increased (Mukherjee et al., 2011; Luque-Casado et al., 2013). Furthermore, HRV was reduced in participants who achieved worse results in cognitive tasks in which the prefrontal area was involved. This could indicate the possibility of HRV predicting cognitive performance (Muthukrishnan et al., 2017), but this association is still unclear in a high cognitive load sport such as chess, where the relation between players’ psychophysiological responses and their performance is currently unknown.

This psychophysiological markers, HRV, has emerged as an interesting tool to monitor training and performance in chess (Fuentes et al., 2018), but also, it could help to determine the psychophysiological response of chess players in cognitive tasks with a different level of difficulty. Traditionally, stress assessment has been easily measured by a visual analogue scale (VAS), and it is considered a valid instrument (Lesage et al., 2012). VAS could allow determining the level of stress of a cognitive task as well as whether the perceived difficulty or complexity of chess problems corresponds with the theoretical difficulty of the problem-solving task or not. These perceptions could reinforce HRV information which may be useful in the interpretation of psychophysiological data.

The present study aimed to evaluate differences between two groups of chess players (divided according to their performance in the high and the low performance group) in the HRV and perceived subjective difficulty, stress and complexity while they were completing six chess problems with different difficulty. The initial hypotheses were that (a) HRV will be reduced and subjective difficulty, stress and complexity will be increased when the difficulty of the problem is increased and (b) players with higher performance will have higher values of HRV and less perceived difficulty, stress and complexity during the different tasks than the low performance group.

## Materials and Methods

### Participants

A total of 16 male chess players, age: 35.19 (13.44) were analyses (see Table 1). All the participants were classified according to the ranking system of the World Chess Federation (FIDE), which was developed by Elo (1978). In the present study, the chess players were divided into two groups, according to the results achieved solving six chess problem tasks: High performance, n: 8; ELO: 1974 (161) or low performance, n: 8; ELO: 1882 (172). The correct solution for each problem-solving task was awarded 1 point. The maximum score was 6. Players who solved more than half of the problem tasks were included in the high performance group. Participants were not on medication that could affect the autonomic nervous system. They gave written informed consent to participate in the study. All procedures were approved by the University of Extremadura research ethics committee (approval number: 85/2015) and were carried out in accordance with the Declaration of Helsinki. Exclusion criteria included: (1) inability to perform the tasks with the computer, (2) diseases that affect the autonomic nervous system, and (3) not being classified by the International Chess Federation with ELO.

**Table 1:** Between group comparisons of the general characteristics, distribution of performance, and basal values of heart rate variability.

Variables	High performance group	Low performance group	*P*-value^a^
**General characteristics**			
Sample Size	8	8	
Age	27.63 (12.05)	42.75 (10.55)	0.038
ELO	1973 (161)	1881 (172)	0.234
**Distribution of performance: number of total correct problems for each group**			
Low level problem	16	14	0.721
Medium level problem	16	7	0.002
High level problem	2	0	0.442
**Heart rate variability measures**		
Mean HR	79.70 (26.23)	78.17 (11.11)	0.959
SDNN	60.05 (15.62)	41.34 (14.94)	0.644
Pnn50	14.21 (15.61)	4.30 (5.23)	0.644
rMSSD	33.29 (19.04)	20.79 (8.80)	0.644
LF/HF	6.80 (8.66)	4.81 (4.43)	1.000
SampEn	1.10 (0.25)	1.16 (0.19)	1.000
SD1	23.59 (13.46)	14.71 (6.24)	0.483
SD2	81.34 (35.04)	56.56 (20.21)	0.798


*ELO: ranking system of the World Chess Federation; HR: Heart Rate; SDNN: Standard deviation of all normal to normal RR intervals; pNN50: The percentage of intervals >50 ms different from preceding interval; RMSSD: Square root of the mean of the squares of successive RR interval differences; LF/HF: ratio Low Frequency (LF) (ms^*2*^)/ High Frequency (HF) (ms^*2*^); SampEn: Sample Entropy; SD1: Dispersion, standard deviation, of points perpendicular to the axis of line-of-identity in the Poincaré plot; SD2: Dispersion, standard deviation, of points along the axis of line-of-identity in the Poincaré plot. ^*a*^Differences between group were detected using Mann–Whitney *U* adjusted by Benjamini–Hochberg procedure.*

In order to calculate the sample size and power analysis of the study, a non-linear measure of HRV known as “SD1” (defined as the long-term beat-to-beat variability using Poincaré plot) was used. The sample size of the current study was 16 participants. This sample size reached 86% power to detect a difference of 14.1 (*p*-value < 0.05) between the null hypothesis and the alternative hypothesis using a Mann–Whitney test.

### Procedure

Before starting the study, participants were given instructions on procedures and protocol requirements during the problem-solving tasks. Participants underwent a familiarization period with the computer and the equipment required for testing. The research was conducted in a laboratory with automatically and continuously controlled temperature and humidity, 20.2 (1.0)°C; 56.4 (2.8)% humidity. Noise levels were kept under 30 dB during all the procedure. All the evaluation was conducted in the morning, between 10:00 and 14:00, with 1 h of no liquid and food ingestion, with no medication or caffeine ingestion in the last 24 h and with at least 24 h since the last vigorous physical activity.

**FIGURE 1:**
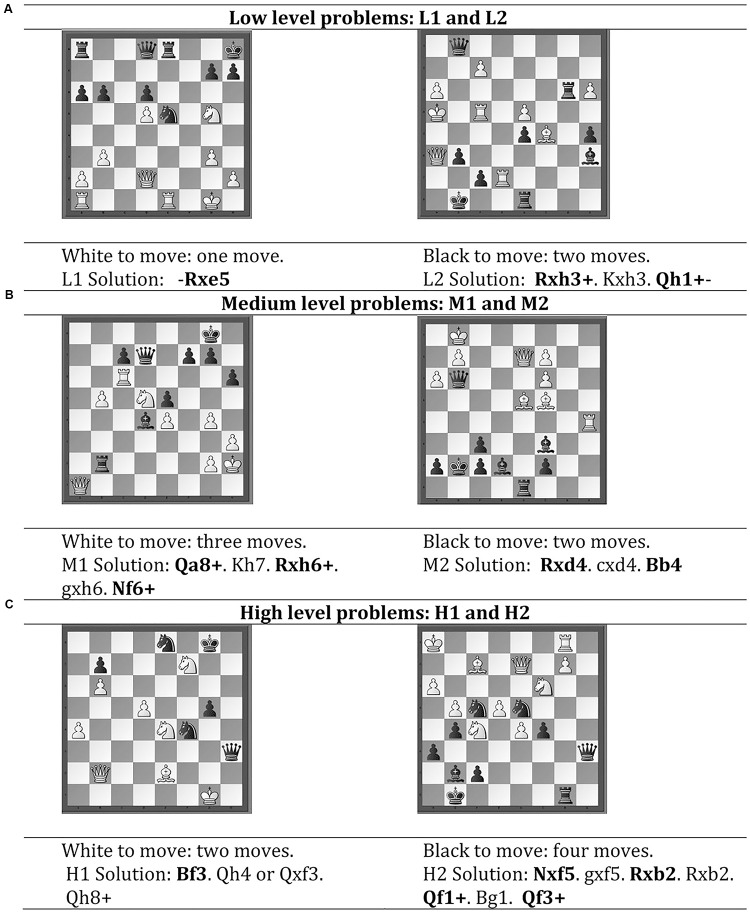
Problem solving tasks for each level of difficulty.

Both groups (high and low performance groups) conducted a total of six chess problem-solving tasks (see Figure 1), which were selected from *Total Chess Training CT-ART 3.0* by a FIDE master (ELO rating of 2300 or more). Chess problems consisted of two low-level, two medium-level and two high-level chess problems (Figure 1). Participants had two and a half minutes to solve each problem. In low-level problems, participants were encouraged to do one move in the first one and two moves in the second problem. In medium-level problems, three moves in the first and two moves in the second problem were required. Finally, in high-level problems participants were asked to do two moves in the first and four moves in the second problem (see Figure 1).

**FIGURE 2:**
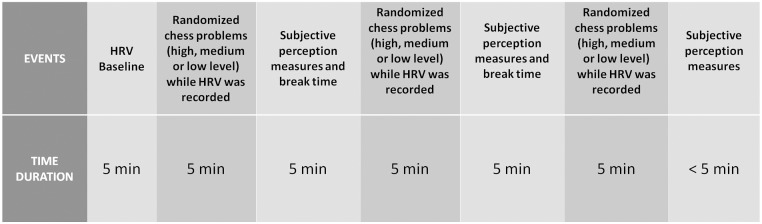
Timeline of the experiment procedures.

Heart rate variability was analyzed at baseline and while participants were completing the problems. Immediately after each level of difficulty (high, medium and low levels), perceived difficulty, stress and complexity of each problem were also measured (see Figure 2). The order of the problems was randomized to avoid the effects of one task on others. Problem-solving tasks were carried out using the 64-bit Fritz 15 chess engine, with Stockfish 6 for Windows. This software is one of the strongest chess engines in the world, and it is open source (GPL license). An ASUS laptop was used (Intel Core i7-6500U, 1 TB, 8 GB memory DDR3L-SDRAM). Fritz software automatically responded to moves, simulating a real chess environment, with the best move previously computed by the research group.

Heart rate variability was recorded at baseline and while performing the tasks, according to the *Task Force of the European Society of Cardiology and the North American Society of Pacing and Electrophysiology* (Camm et al., 1996) during a short-term 5-min period in sitting position and also following the instructions of previous studies (Beltrán-Velasco et al., 2018; Sánchez-Molina et al., 2018). HRV was measured with a reliable heart rate monitor (Polar RS800CX, Finland) (da Costa et al., 2016). Time and frequency domains, as well as non-linear measures, were extracted using Kubios HRV software (v. 2.1) (Tarvainen et al., 2014). HRV measures were merged for each level of difficulty. The duration of each level of difficulty was 5 min to comply with the short-term HRV recording recommendation (Camm et al., 1996). A medium filter was applied to correct artifacts, and the correction level identified all beat to beat intervals (RR) that were longer/shorter than 0.25 s compared to the local average. The correction was made by replacing the identified artifacts with interpolated values using a cubic spline interpolation.

For the time domain, the heart rate (HR), the standard deviation of all normal to normal RR intervals (SDNN), the percentage of intervals >50 ms different from preceding interval (pNN50) and the root of the mean of the squares of successive RR interval differences (RMSSD) were analyzed. In the frequency domain, the ratio between Low Frequency (LF) (ms^2^)/ High Frequency (HF) (ms^2^) was calculated. On the other hand, non-linear measures such as the Sample Entropy (SampEn) and the dispersion, standard deviation, of points perpendicular to the axis of line-of-identity in the Poincaré plot (SD1) and the dispersion, standard deviation, of points along the axis of line-of-identity in the Poincaré plot (SD2) were also included in the analysis. Higher values of SDNN, RMSSD, SD1, and SampEn, are associated with parasympathetic modulation as well as a reduction in these previous indexes or lower values of LF/HF and SD2 are associated with sympathetic modulation (Kamen et al., 1996; Karmakar et al., 2011; Soares-Miranda et al., 2014; Weippert et al., 2014).

A Visual Analogue Scale (VAS) (0–10) was used to measure perceived stress (during the problem-solving tasks), difficulty (perceived difficulty for each problem) and complexity (subjective perception of reaching the goal, taking into account both the limited period of time and game difficulty) after every single task. These behavioral data were collected to reinforce HRV information and to provide useful information for the interpretation.

### Statistics

The SPSS statistical package (version 20.0; SPSS, Inc., Chicago, IL, United States) was used to analyze the data. Considering the sample size (*n* = 16) and the results in the Shapiro–Wilk and Kolmogorov–Smirnov tests, non-parametric analyses were conducted.

Friedman’s ANOVA by ranks was used to evaluate within-group differences in HRV and subjective perception in the three conditions: low, medium and high difficulty levels. Adjusted post-hoc with Dunn-Bonferroni for multiple comparisons was obtained to control Type I error (Dunn, 1961). Differences between these two groups for each level of difficulty were assessed using the Mann–Whitney *U*-test. The alpha-level of significant (set at 0.05) was adjusted by Benjamini–Hochberg procedure in order to control the false discovery rates (Benjamini and Hochberg, 1995).

## Results

Participants were classified according to their performance on the tasks into two groups: high and low performance groups. Mann–Whitney *U* at baseline revealed significant differences between groups at age (*U* = 12.50, *p* = 0.038, *r* = 0.51), with high performance group aged 27.63 (12.05) and low performance group aged 42.75 (10.55). However, non-significant differences between groups were observed at baseline in any of the HRV measures or ELO (*U* = 20, *p* = 0.234, *r* = 0.31). The high performance group achieved a significantly higher number of correct solved problems in the medium level problem [χ^2^ (1, *N* = 16) = 12.44, *p* < 0.01] (see Table 1).

### Within-Group Differences

In the high performance group, the task difficulty increment led to significant changes in the autonomic modulation and subjective difficulty, stress and complexity. Comparing medium vs. high-level problems, the HRV (RMSSD and SD1) significantly decreased when difficulty increased. In the same line, when compared low vs. high-level problems, subjective difficulty, stress and complexity increased (see Table 2). In the low performance group, significant effects of the difficulty increments were observed in mean heart rate and perceived difficulty and stress when low vs. high-level problems were compared (see Table 2).

**Table 2:** Mean (SD) of the different chess problem for each group and intra and inter groups comparisons for heart rate variability and subjective perception.

	High performance group			Low performance group								
	Low level problem	Medium level problem	High level problem	Low level problem	Medium level problem	High level problem	Within group differences^a^			Differences between low and high performance groups^b^		
							High performance group		Low performance group			
									
							Low vs. high level	Medium vs. high level	Low vs. high level	Low level	Medium level	High level
**Heart rate variability**		
Mean HR	78.16 (14.09)	78.79 (15.71)	80.55 (13.70)	82.20 (11.44)	84.10 (11.20)	87.00 (11.07)	ns	ns	0.037	ns	ns	ns
SDNN	70.58 (18.09)	68.18 (17.24)	67.28 (25.03)	44.36 (12.74)	46.45 (15.88)	44.10 (17.02)	ns	ns	ns	0.021	0.027	ns
Pnn50	18.03 (14.68)	19.44 (17.06)	15.46 (15.92)	3.76 (4.01)	5.00 (7.14)	3.65 (4.49)	ns	ns	ns	0.021	0.027	ns
RMSSD	40.69 (19.02)	42.01 (21.12)	36.65 (20.37)	20.65 (8.06)	21.55 (9.48)	18.61 (8.64)	ns	0.037	ns	0.021	0.027	ns
LF/HF	4.12 (2.42)	4.48 (2.62)	3.95 (2.05)	5.81 (3.73)	5.83 (3.77)	5.51 (4.90)	ns	ns	ns	ns	ns	ns
SampEn	1.18 (0.42)	1.19 (0.34)	1.19 (0.22)	1.04 (0.32)	1.08 (0.25)	0.90 (0.25)	ns	ns	ns	ns	ns	0.041
SD1	28.81 (13.47)	29.76 (14.95)	25.94 (14.42)	14.76 (5.66)	15.25 (6.73)	13.16 (6.12)	ns	0.037	ns	0.032	ns	0.027
SD2	95.08 (23.56)	78.26 (33.07)	91.43 (32.77)	61.56 (16.95)	63.76 (21.79)	60.89 (23.40)	ns	ns	ns	0.032	ns	ns
**Subjective perception**							
Stress	1.75 (1.83)	2.29 (1.60)	5.38 (2.72)	2.25 (1.58)	4.13 (2.30)	6.12 (2.80)	0.004	ns	0.005	ns	ns	ns
Difficulty	1.75 (1.75)	3.71 (1.70)	8.13 (0.83)	3.25 (2.38)	5.38 (1.69)	7.75 (1.16)	0.002	ns	0.012	ns	ns	ns
Complexity	0.38 (1.06)	0.86 (0.69)	6.00 (2.56)	4.25 (3.92)	5.88 (2.75)	6.88 (1.96)	0.006	ns	ns	0.018	0.009	ns


*ns: Non-significant; HR: Heart Rate; SDNN: Standard deviation of all normal to normal RR intervals; pNN50: The percentage of intervals >50 ms different from preceding interval; RMSSD: Square root of the mean of the squares of successive RR interval differences; LF/HF: ratio Low Frequency (LF) (ms^*2*^)/High Frequency (HF) (ms^*2*^); SampEn: Sample Entropy; SD1: Dispersion, standard deviation, of points perpendicular to the axis of line-of-identity in the Poincaré plot; SD2: Dispersion, standard deviation, of points along the axis of line-of-identity in the Poincaré plot. ^*a*^Intra group significant differences were detected using Friedman’s ANOVA by ranks. *P*-value was corrected using Dunn-Bonferroni adjustment. Only significant interactions between chess problems were reported. ^*b*^Differences between low and high performance groups in each difficulty level were detected using Mann–Whitney *U*-test. The alpha level of significance was adjusted by Benjamini–Hochberg procedure.*

### Between-Group Differences

During low-difficulty level problems, the high performance group obtained an increased HRV (higher values of SDNN, Pnn50, rMSSD, and SD1) and a reduction in the perceived complexity compared to the low performance group (see Table 2). In the same line, in the medium-difficulty level problem, the high performance group obtained higher values of HRV (higher values of SDNN, Pnn50, and rMSSD) and a reduction in the perceived complexity compared to the low performance group (see Table 2). Moreover, during the high-level problems, higher parasympathetic activity (higher values of HRV variables such as SampEn and SD1) were found in the high performance group compared with the low performance group while the perceived complexity was not different (see Table 2).

## Discussion

The present study examined differences in HRV and perceived subjective difficulty, stress and complexity during chess tasks of varying difficulty in different chess performance players. Overall, results showed that when task difficulty increased, HRV decreased in both the high and the low performance groups. Regarding subjective measures, perceived difficulty, stress and complexity increased when the difficulty of the task increased in the high performance group, whereas in the low performance group, only perceived difficulty and stress increased when the task difficulty increased. Therefore, results were in line with the initial hypotheses, where we stated that highly difficult tasks would produce a decrease in HRV. That hypothesis assumed that higher-performing players would reach higher parasympathetic modulation (higher HRV), as well as lower perceived stress, difficulty and complexity during the different tasks than the low performance group.

Within-group results in terms of HRV indicated that SD1 and rMSSD were higher in the medium level problems compared with the high level problems in the high performance group. This could indicate that the sympathetic responses were higher when the problem difficulty increased, which could be aimed to deal with the problem’s cognitive demands (Wickens et al., 2015). In line with these results, previous researchers have shown that the greater the cognitive load, the lower the HRV (Hjortskov et al., 2004; Mukherjee et al., 2011), supporting the idea of HRV as a reliable and sensitive index to mental effort (Mukherjee et al., 2011). In addition, an increased in the subjective perception of stress, difficulty and complexity was observed between the low and the high level problems. However, the low performance group did not show these patterns in HRV when the task difficulty was increased. This could indicate that the cognitive requirements of the three problems were too high for them. This hypothesis could be supported by the differences in the perception of complexity showed in the between group comparisons. In this regard, the low performance group perceived both the low and the medium difficulty levels as more complex compared with the high performance group. Therefore, HRV seems to be sensitive enough to be used as a training tool for monitoring and controlling the cognitive load.

In addition, between-group analysis revealed that HRV – specifically SDNN, rMSSD and Pnn50 – was significantly higher in the high performance group than in the low performance group during chess problems of varying difficulty. The unsuccessful performance of the low performance group could be related to the higher anxiogenic response, associated to the significantly lower HRV values, in comparison with the high performance group (Gur et al., 1988). In line with this result, a previous study reported that HRV was reduced in a group who achieved worse results in a cognitive task (Muthukrishnan et al., 2017). Findings from the current study could support the possibility of HRV predicting cognitive performance in chess players.

Chess has been used for the study of cognitive processes such as memory, problem solving or decision making (Chase and Simon, 1973; Kiesel et al., 2009; Villafaina et al., 2018). In our study, different chess problem tasks where abilities like logic and mathematics have a great influence were proposed. Trinchero and Sala (2016) hypothesized that the heuristic method, i.e., a method for arriving at satisfactory solutions with the modest amount of computations (Simon, 1990), could help to reduce the potential solutions of the task and lead to a reduction in the cognitive load (Shah and Oppenheimer, 2008). In the current study, higher values of HRV were detected in the high performance group compared with the low performance group, which could indicate that high performance group experimented lower cognitive load. Future studies are encouraged to explore the potential association of cognitive load and HRV following the basis of the heuristic method.

Chess has been used for the study of cognitive processes such as memory, problem-solving or decision making (Chase and Simon, 1973; Kiesel et al., 2009; Villafaina et al., 2018). In our study different chess problem tasks, where abilities like logic and mathematics have a great influence, were proposed. Trinchero and Sala (2016) hypothesized that the heuristic method, i.e., a method for arriving at satisfactory solutions with the modest amount of computations (Simon, 1990), could help to reduce the potential solutions of the task and lead to a reduction in the cognitive load (Shah and Oppenheimer, 2008). This hypothesized reduction in the cognitive load could lead to a reduction in the sympathetic modulation, decreasing the HRV indexes. We reported in our study higher values of HRV in the high performance group compared with the low performance group while performing different levels of problem difficulty. Probably, this could indicate that the cognitive load of the high performance group was lower. Therefore, it is possible that this group had more problem-solving heuristic resources than the low performance group. However, future studies should evaluate this hypothesis.

Results of within and between group analyses revealed significant differences in SD1 (an HRV non-linear measure). This could indicate that this index may be more sensitive to changes caused by cognitive processing than others. In line with our results, time measures and SD1 have emerged as reliable and sensitive indexes of mental effort (Mukherjee et al., 2011). This may support the idea of non-linear analysis as an adequate measure in complex dynamic systems (Goldberger, 1996) and reinforce the suitability of HRV to be used to monitor and control the mental effort.

One limitation of the current study was the significant between-group differences that were observed in the participants’ age. However, the two groups did not statistically differ in HRV at baseline in any of the studied variables. Nevertheless, since HRV could be influenced by age (Reardon and Malik, 1996) and there is a lack of studies focused on the influence of age on HRV during cognitive tasks, the obtained results should be taken with caution. Also, the sample size was relatively small. Another potential limitation may be the fact that problem-solving tasks were conducted with the computer. This could affect motivation, and therefore the cognitive engagement of our participants in the problem-solving tasks. Although all participants had played chess with the computer previously, results might be different to those obtained in “real” chess. Furthermore, the sample size (*n* = 16) was relatively small, which could also affect the HRV parameter, making harder the comparisons according to body mass index or age. This study was only developed with men, so future studies should determine if these results could be extrapolated to women.

### Practical Applications

Results of the present study showed how increasing the difficulty level of chess problems could modify the HRV according to cognitive performance. Thus, HRV is sensitive enough to detect changes in HRV as a consequence of the increase of the cognitive demands.

Therefore, monitoring the HRV of chess players could be useful to control the cognitive load of the proposed tasks. Although further research is needed, when the HRV of the chess player is decreased, the cognitive stimulus may be leading to a relevant cognitive load. However, when the cognitive load does not change the HRV of the player, probably the cognitive load may be too high or too low (as could happen in the low performance group). Thus HRV is presented as an easy and highly applicable training tool for chess players that may be considered by trainers to control the cognitive load in chess-related tasks.

## Conclusion

To conclude, this is the first study reporting a decrease of HRV to meet the cognitive demands of the problems in chess players. HRV was significantly higher in the high performance group than in the low performance group when solving chess problems of varying difficulty. In addition, the low performance group perceived the problem solving tasks as more complex than the high performance group. These results open a new field where HRV could be an interesting and useful tool in chess training to assess the cognitive demands and capacities of chess players. However, the relatively small sample size and the difference in age between groups makes that these findings should be taken with caution.

## Author Contributions

JF-G, SV, and VC-S conceived the study. JF-G, SV, and DC-M collected the data. PO, VC-S, RdV, DC-M, JF-G, and SV analyzed the data. PO, VC-S, RdV, and DC-M designed the figures and tables. SV, JF-G, and DC-M wrote the manuscript. VC-S, SV, PO, and RdV provided critical revisions on the successive drafts. All authors approved the manuscript in its final form.

## Conflict of Interest Statement

The authors declare that the research was conducted in the absence of any commercial or financial relationships that could be construed as a potential conflict of interest.
